# Quality of nursing work life, job satisfaction, and intent to leave among Jordanian nurses: A descriptive study

**DOI:** 10.1016/j.heliyon.2022.e09838

**Published:** 2022-07-01

**Authors:** Muna Fayez Salahat, Zaid Mohammed. Al-Hamdan

**Affiliations:** Department of Community and Mental Health Nursing, Faculty of Nursing, Jordan University of Science and Technology, Irbid, Jordan

**Keywords:** Nursing work life, Quality, Job satisfaction, Intent to leave, Nursing, Jordan

## Abstract

**Background:**

Nurses’ intentions to leave their job are considered among the several difficulties encountered by any health system. Quality of nursing work life (QNWL) and job satisfaction are among the significant factors that impact this intention.

**Purpose:**

To explore the relationship between quality of work-life job satisfaction and the intention to leave among registered nurses (RNs) in Jordanian hospitals.

**Methods:**

A cross-sectional study using a self-administered questionnaire was conducted to collect data from 200 RNs from three hospitals in Jordan to measure the main variables from July 11 to 25,2020. Data were analyzed using descriptive and inferential statistics using SPSS version 25.

**Results:**

A total of 193 RNs participated in the study and were moderately satisfied with their quality of work life and job satisfaction. QNWL correlated positively with job satisfaction (*r* = 0.579, p = 0.000) and negatively with intent to leave (*r*_*s*_ = - 0.204, p-value = 0.002). Job satisfaction was negatively correlated with the intent to leave (*r*_*s*_ = - 0.174, p-value = 0.008). Sociodemographic and work-related variables (hospital type, sex, educational level, and salary) showed significant differences and correlations with at least one of the main variables.

**Conclusion:**

This study's results can be used by health care managers and policymakers to implement successful plans and policies to improve the QNWL and job satisfaction of RNs. This, in turn, may assist in enhancing individual and organizational performance; improving home and job environments; increasing nurses' commitment and increasing nursing retention. Further research is needed to develop effective measures for enhancing QNWL and job satisfaction among RNs.

## 1 Introduction

Undoubtedly, that the largest workforce in the healthcare system is nurses, with an estimated 19.3 million nurses out of a total of 43.5 million health workers worldwide (World Health Organization, 2020) [1]. Thus, the quality of hospital services and patient care cannot be improved without the contribution of this workforce [2].

There is an increasing interest in improving nurses' working environments [3]. As such, numerous organizations strive to empower their staff's abilities to provide a coherent environment and improve their synergetic forces. Creating such an environment is said to be a step towards improving the quality of work life [4].

QNWL is defined as “The degree to which registered nurses are able to satisfy important personal needs through their experiences in their work organization while achieving the organization's goals” (Brooks, 2001, p. 9) [5].

QNWL is associated with several positive consequences, such as improving the health system's productivity and efficiency [6], improving performance [7], increasing employee empowerment [8], and reducing employee turnover [9]. In contrast, poor QNWL leads to increased turnover intention [10]; increased stress, which affects the stability and management of any organization [11], obstructs workflow in the organization, and is an obstacle to entry into nursing [12].

Regular evaluation of work-life quality can afford organizations with significant knowledge about the aspects of employee life at all levels [13].

QNWL is considered a comprehensive department-level program dedicated to improving employee satisfaction, enhancing learning in the workplace, and assisting employees in better managing change and transition [14].

According to McCloskey and McCain [15], job satisfaction is; the level at which workers enjoy their jobs.

Job satisfaction is considered to be a global concern; however, it is also essential to improve the quality of care delivered and cultivate an appropriate work environment in healthcare organizations [16], and the absence of job satisfaction among nurses can affect their practice, which in turn can affect patients' satisfaction directly or indirectly [17]. Low job satisfaction is considered a contributing factor to nurses leaving their current jobs and profession [18]. Intent to leave is considered to be the most accurate predictor of nurse turnover and actual leaving [19], and it can be defined according to Weisberg [20] as; the employee's intention to plan to leave his current job in order to find another one that he looks forward to in the near future.

Several healthcare organizations encounter shortages of healthcare providers and an increase in turnover rates, especially among nurses [21]. Jordan, like many other countries, suffers from a high turnover rate among nurses, which reached about 36.6% [22]. This has a negative impact on the patient's care and increases mortality and infection rate [23]. The ongoing nursing shortage and consequent high rate of nurse turnover are considered critical challenges for healthcare organizations and affect several domains, from the quality of nursing services to the organization's productivity [24].

Retaining qualified nurses is critical for the survival of health organizations [25]. One of the most promising ways to understand and explore the recruitment and retention of nurses is to assess the quality of work life and related factors [26, 27]. This study aimed to explore the relationship between QNWL, job satisfaction and intention to leave among Jordanian nurses. This was achieved through the following research questions:

1. What is the relationship between QNWL and job satisfaction?
2. What is the relationship between QNWL and the intent to leave?
3. What is the relationship between nurses' job satisfaction and the intent to leave?
4. What are the differences in QNWL, job satisfaction, and intent to leave according to nurses' sociodemographic variables?
5. What are the relationships between QNWL, job satisfaction, intent to leave, and sociodemographic variables?

## 2 Materials and methods

### 2.1 Study design

A descriptive cross-sectional correlational design was used, because it assists in determining the interrelationship between the study variables within a short period of time during the study [28]. Additionally, the direction, strength, and significance of the relationships between the study variables were acceptable, as measurements were collected only once during the study. Furthermore, the data acquired can be utilized in different types of research, and the results can be reanalyzed to create in-depth research or new studies in the future.

### 2.2 Setting and participants

A non-random method of hospital selection was employed by including Jordanian hospitals with more than 200 beds from the following healthcare sectors: a governmental hospital with a 1100 bed capacity; a private hospital with a 280- bed capacity, both located in the capital of Jordan; and a teaching hospital located in the north of Jordan with a bed capacity of 538. All the targeted hospitals provided healthcare services for all specialties and fields.

Concerning the study sampling, the target population was all Jordanian RNs, while the accessible population was Jordanian RNs working in the targeted hospitals. The sample size was calculated using G∗ power, where power = 0.80, medium effect size, α = 0.05, which resulted in 180 participants, which was then increased by 10% for non-respondents and probable missing data. The final sample size of N = 200 RNs was selected using a non-probability convenient sampling technique. The inclusion criteria were as follows: being a registered nurse working full time, and providing bedside care in one of the departments that operates on a 24- hours basis. The exclusion criteria were licensed practical nurses and RNs who worked in administrative positions such as charge nurses, nurse managers, and RNs in all departments that do not operate for 24 h. One hundred and ninety-three participants out of 200 completed the questionnaires, with a response rate of 96.5% during the period of July 11 to 25 2020 targeting the three working shifts.

### 2.3 Ethical considerations

#### 2.3.1 Ethical approval was obtained from the institutional

Review Board Committee of the Jordan University of Science and Technology and the hospitals where the data were collected. Written permission to use the study instruments was obtained from the authors by e-mail. Nurses were invited to participate in the study after obtaining informed consent. Raw data were destroyed after the completion of the study.

### 2.4 Instruments

The study questionnaire was adapted from Brooks' QNWL survey [29], Muller and McClosky satisfaction scale [30], and demographic information was added. Approval to use the original copyrighted material from the QNWL survey was obtained from the original author. For the Arabic version, see Almalki's paper [12]. The Muller and McClosky satisfaction scale permission was obtained from the author for the validated Arabic version, where the alpha coefficient was 0.83 [31]. Furthermore, a pilot study was conducted with 20 participants to determine the clarity and reliability of the instruments used and estimate the time required to complete them.

#### 2.4.1 Demographic information sheet

The demographic information sheet contained 14 items to collect data on selected demographic variables such as age, years of experience, department, and qualifications.

#### 2.4.2 Brooks’ quality of nursing work-life scale

The QNWL scale was originally developed to measure work-life quality among RNs [29], which contains 42 items divided into four dimensions:” home life/work life” (7 items) refers to the interface between nurses' life experiences at their workplace and at home; the second dimension “work design” (10 items) refers to nursing work composition and describes the actual work performed by the nurses; the third dimension “work context” (20 items) includes the practice settings in which nurses work and discovers the work environment impact on the nurse and patient systems; and the fourth dimension “work world” (5 items) considers the impacts of broad societal effects and changes on nursing practice. Table 2 presents the dimensions of the QNWL. To indicate the levels of QNWL, Brooks set a cut-off point for the total score as follows: low (42–112), moderate (113–182), and high (183–252). QNWL was assessed on a 6-point Likert scale ranging from 1 = strongly disagree to 6 = strongly agree. The minimum total score on the scale is 42 and the maximum is 252, which indicates a high QNWL. According to Brooks, the Cronbach's alpha coefficient, which estimates the instrument reliability ranges from 0.56 to 0.88, was considered reliable and valid [29]. In this study, the reliability of the instrument estimated using Cronbach's alpha was 0.91.

#### 2.4.3 Mueller and McClosky Satisfaction Scale [MMSS]

Developed by Muller and McClosky [30], this scale contains 31 items divided into eight subscales which are as follows: family/work balance, 3 items (7, 11, 12); interaction, 4 items (16, 17, 18, 19); scheduling, 6 items (4, 5, 6, 8, 9, 10); extrinsic rewards, 3 items (1, 2, 3); praise and recognition, 4 items (13, 24, 25, 26); work control and responsibility, 5 items (22, 23, 29, 30, 31); professional opportunities, 4 items, (20, 21, 27, 28); and co-workers, 2 items (14, 15). For each subscale, the scores are summed and divided by the number of items to obtain the mean, where mean less than 3 indicates dissatisfaction; equal to three indicates neutral; and more than 3 indicates satisfaction. Table 3 presents the MMSS and its subscales. The MMSS is assessed on a 5-point Likert scale ranging from 1 = (very dissatisfied) to 5 = (very satisfied). The scale exhibits high reliability and validity, and the overall alpha coefficient for the original scale is 0.89. In this study, the reliability of the instrument, estimated using Cronbach's alpha, was 0.93.

#### 2.4.4 Intent to leave

Intent to leave was measured with a single question — “Do you intend to leave your job within the next 12 months?” —added to the demographic information sheet and employed a 5-point scale (1 = very likely; 2 = likely; 3 = neutral; 4 = unlikely; 5 = very unlikely); if the answer was yes, we asked the respondents to explain why.

### 2.5 Data analysis

To address the research questions; SPSS version 25 was used to analyze the data at an alpha level of 0.05 for all statistical tests. Descriptive statistics (means, frequencies, standard deviations and percentages) were analysed for all variables and demographic data.

Several tests were selected according to the normality test (Kolmogorov–Smirnov test), and the Mann-Whitney test was used to test the difference in ranks of scores between the two groups. An independent sample t-test was used to test the difference in means between the two groups. While for three or more groups, a one-way analysis of variance (ANOVA) test was used, a post-hoc (Tukey) test was utilized to determine the source of the difference, and Pearson's and Spearman's correlation tests were used to test the relationships between the main variables and sociodemographic and work-related variables.

## 3 Results

### 3.1 Sample characteristics

Out of the 200 questionnaires distributed to RNs in targeted hospitals, 193 completed the questionnaire with a 96.5 % response rate. The study results showed that the mean age of the participants was 32.9 years (ranging from 22 to 48 years, SD = 6.029). Most participants were female (65.8%, n = 127), who were married (66.6%, n = 143), with children (69.40%, n = 134). They held bachelor's degrees (86%, n = 166). Their years of experience in nursing ranged from 1 to 22 years ($\bar{X}$ = 8.67, SD = 5.38); half of them (50.8%, n = 98) indicated that their salary was within the average salary range in Jordan, which is equal to 500 Jordanian dinars. Also, 84.5% (n = 163) worked rotating shifts, and 85% (n = 164) were not certified in the specialty area in which they worked, i.e., had no additional certificates other than their original educational level. Table 1 presents the sample characteristics.

**Table 1:** Sociodemographic and work-related characteristics of the participants (N = 193).

Variable	N	Frequency%	Mean	SD
**Age**				
Minimum = 22 years	193	----	32.69	6.029
Maximum = 48 years				
**Gender**				
Male	66	34.2	----	----
Female	127	65.8		
**Marital Status**				
Single	44	33.4	----	----
Married	143	66.6		
Divorced	5	2.6		
Widowed	1	0.5		
**Highest degree of education in nursing**				
Bachelor's degrees	166	86.0	----	----
Master's degrees	27	14.0		
**Dependent children**				
No	58	30.1	----	----
Yes	134	69.4		
**Dependent Adult**				
No	95	49.2	----	----
Yes	98	50.8		
**Years of experience in nursing as RN**				
Minimum = 1 year	193	----	8.67	5.38
Maximum = 22 years				
**Monthly salary compared with average salary in Jordan**				
Lower than average	48	24.9	----	----
Within average	98	50.8		
Higher than average	47	24.4		
Hospital type				
Governmental	64	33.2	----	----
Private	64	33.2		
University	65	33.7		
**Type of unit where you currently work**				
Emergency	32	16.6	----	----
Medical	26	13.5		
Surgical	34	17.6		
Adult ICU & CCU	35	18.1		
PICU & NICU	14	7.3		
Paediatric	11	5.7		
Obstetrics & gynecology	9	4.7		
Operation	32	16.6		
**Rotate shift**				
No	30	15.5	----	----
Yes	163	84.5		
**Certification in specialty area**				
No	164	85	----	----
Yes	29	15		
**Additional compensation for being certified**				
No	189	97.9	----	----
Yes	4	2.1		
**Intention to leave job within the next 12 months**				
Strongly likely	15	7.8	----	----
Likely	30	15.5		
Neutral	35	18.1		
Unlikely	59	30.6		
Strongly unlikely	54	28		
**Causes of intent to leave**				
Low salary	13	5.2	----	----
Looking for better opportunity	7	3.6		
Travel	7	3.6		
Workload	3	1.6		
Complete study	2	1.0		
Family causes	2	1.0		
Early retirement	1	0.5		
Instability and changes in system	1	0.5		
No annual increase	2	1.0		
Physical & psychological stress	1	0.5		
Unfair among staff	2	1.0		
Profession doesn't suit his/her personality	1	0.5		
Looking for more comfortable place	3	1.6		

### 3.2 Quality of Nursing Work Life

With respect to QNWL in the current study, RNs scored 59 to 218 in the actual range, with a total mean score of 158.55 for the whole scale, which is higher than the average score on the Brooks scale (147), indicating a moderate QNWL. The major influencing factors were categorized into four dimensions.

First, “home life/work life” factors: no energy left after work (84.5%, n = 163); system of working hours negatively impacts their life (69.9%, n = 135); inadequacy of vacation policy (59.1%, n = 114); need to have onsite/near childcare services (87.6%, n = 169); need for ill childcare services (85.5%, n = 165); need for day care of elderly parents’ services (79.8%, n = 154).

Second, work design factors: high workload (88.1%, n = 170); not enough RNs (66.8%, n = 129); time to do jobs (59.6%, n = 115); undertaking many non-nursing tasks (69.9%, n = 135); encountering many interruptions during daily work routines (65.8%, n = 127); and lack of autonomy (51.3%, n = 99).

Third, work context factors: lack of opportunities for career advancement (58%, n = 112); no

participation in decision making (57%, n = 110); unavailability of break areas for nurses (89.6%, n = 173); and nursing degree granting programs (67%, n = 130).

Finally, work world factors: inadequate payment (67.4%, n = 130) and a negative public image of nursing (62.2%, n = 120).

In general, RNs were more satisfied in the work context dimension ($\bar{X}$ = 77.81)and less satisfied in the work world dimension ($\bar{X}$ = 17.52). Table 2 presents the QNWL scale and the mean scores of its dimensions.

**Table 2:** Total scale scores and dimensions scores for QNWL survey.

Scale	possible range	Average	Mean (SD)	Actual range
42 item scale	42–252	147	158.55 (SD = 25.49)	59–218
Homelife/work life (7 items)	7–42	24.5	25.56 (SD = 4.28)	8–38
Work design (10 items)	10–60	35	37.69 (SD = 6.75)	14–56
Work context (20 items)	20–120	70	77.81 (SD = 14.64)	29–118
Work world (5 items)	5–30	17.5	17.52 (SD = 3.50)	7–25

**QNWL**: Quality of Nursing Work Life.

### 3.3 Job satisfaction

The total mean was 99.88 (SD = 20.93), indicating that the participants were moderately satisfied with their jobs. Regarding the MMSS subscales, the highest mean score was for co-workers with $\bar{X}$= 3.87 (SD = 0.77), while the lowest mean score was for extrinsic reward, $\bar{X}$= 2.84 (SD = 1.08). Table 3 presents the total MMSS and its subscale scores.

**Table 3:** Total MMSS and subscales scores.

Scale	Mean	SD
31 item scale	99.88	20.93
Co-workers (2 items)	3.87	0.77
Interaction (4 items)	3.63	0.74
Praise/Recognition (4 items)	3.36	0.94
Control/Responsibility (5 items)	3.36	0.84
Professional opportunities (4 items)	3.03	0.86
Scheduling (6 items)	2.98	1.01
Family/Work balance (3 items)	2.90	0.96
Extrinsic Reward (3 items)	2.84	1.08

MMSS: Mueller & McClosky Satisfaction Scale.

The RNs in the current study were “moderately satisfied” ($\bar{X}$ > 3) in terms of: **1)** co-workers— such as nursing peers and the physicians they work with; **2)** interaction—such as the social contact with their colleagues after work, care delivery method used in the unit, and opportunities for social contact at work and for professional interactions with other disciplines; **3)** praise/recognition— such as recognition from immediate supervisor, work peers, and work supervisor, and the amount of encouragement and positive feedback; and **4)** control/responsibility— such as the opportunities for career advancement, control over occurrences in the work setting, amount of responsibility, participation in organizational decision making, and control over work.

The RNs were neutral ($\bar{X}$ = 3) in the professional opportunities subscale, such as opportunities to interact with the faculty of the College of Nursing, participation in nursing research, academic writing and publishing, and taking roles in department and institutional committees.

The RNs reported that they were “moderately dissatisfied” ($\bar{X}$ < 3) with the following: **1)** scheduling— such as the number of working hours, working straight days, flexibility in scheduling work hours, number of weekends off per month, scheduling weekends off, and compensation for working on weekends; **2)** family/work balance chances—childcare facilities, maternity leave time, and opportunity for part-time work; and **3)** extrinsic rewards— benefits package (insurance, retirement), salary, and vacation.

### 3.4 Intent to leave

With respect to intent to leave, RNs who intend to leave their jobs within the next 12 months were 23.3% (n = 45), while 58.5% (n = 113) did not intend to leave their jobs, and 18.1% (n = 35) were neutral.

The participants reported the following reasons for their intent to leave: low salary: looking for better opportunities; workload; pursuing higher education; family issues; early retirement; instability and changes in the system; travel, physical and psychological stress; unfair treatment; no annual increase in salary; looking for a more comfortable place; and finally, the profession does not suit his/her personality. Table 1 shows the intent to leave the reasons with the participants’ data.

### 3.5 Correlation between the main variables

Regarding the relationship between QNWL and job satisfaction, the results of the Pearson's correlation test indicated a strong and significantly positive correlation (191) (r = 0.579, p = 0.000). However, Spearman's correlation test revealed a weak and significantly negative relationship between QNWL and intent to leave; *r*_*s*_ (191) = -0.204, p = 0.002, Regarding the relationship between job satisfaction and intention to leave, the correlation was weak and significantly negative *r*_*s*_(191) = - 0.174, p-value = 0.008. Table 4 shows the correlations between the main variables according to sociodemographic characteristics.

**Table 4:** Correlation between the main variables according to sociodemographic characteristics.

Variable	QNWL			Job satisfaction		Intent to leave	
	*r*_*s*_	Sig.		*r*_*s*_	Sig.	*r*_*s*_	Sig.
Gender	0.125∗	0.04		0.059	0.20	-0.15∗	0.01
Age	-0.015	0.41		0.022	0.38	0.035	0.31
Highest education Degree in nursing	0.010	0.44		0.126∗	0.041	0.164∗	0.01
Marital status	-0.058	0.21		-0.032	0.33	0.107	0.70
Dependent children	-0.075	0.14		-0.068	0.17	0.11	0.06
Dependent adult	0.066	0.18		-0.003	0.48	0.012	0.43
Salary	0.024	0.37		-0.041	0.28	-0.139∗	0.02
Hospital type	0.197∗∗	0.003		0.119∗	0.05	-0.113∗	0.05
Unit type	0.015	0.41		0.034	0.31	0.013	0.42
Rotating shift	-0.049	0.25		-0.043	0.27	0.008	0.45
Experience years	0.027	0.35		0.006	0.46	0.085	0.11
Certified in specialty area	0.044	0.27		-0.009	0.44	0.042	0.27
Additional compensation	-0.016	0.41		-0.024	0.37	0.082	0.12

**Sociodemographic characteristics**	**r**	**Sig.**		**r**	**Sig.**	**r**	**Sig.**

**Number of children**	-0.001	0.49		0.019	0.39	0.059	0.20

∗ Correlation is significant at the 0.05 level (1 tailed).

∗∗ Correlation is significant at the 0.01 level (1 tailed).

### 3.6 The differences between the main variables according to nurses’ sociodemographic and work-related variables

Inferential statistics results indicated that participants in governmental hospitals had significantly lower QNWL (F (2, 190) = 11.1, p < .001) and job satisfaction (F (2, 190) = 6.56, p < .001), and higher intent to leave (F (2, 190) = 4.00, p = 0.02) than those in university and private hospitals. Additionally, the Mann–Whitney U test indicated that females reported better QNWL scores than males. U = 3556; N_1_ = 66; N_2_ = 127; p = 0.04.

Furthermore, the study participants who held master's degrees were significantly less satisfied with their jobs (t (191) = 1.60, p = 0.05 (one-tailed)) and expressed significantly higher intent to leave (t (191) = - 1.98, p = 0.02 (one-tailed)).

Additionally, participants whose salary was lower than average had a significantly higher intent to leave (F (2, 190) = 3.96, p = 0.02) than those with higher than average salary (p < .001) and average salary (p < .001). Finally, the intent to leave was significantly lower for female participants than for male participants (t (191) = 2.13, p = 0.01). However, no statistically significant differences were found in the main variable scores with regard to sociodemographic and work-related variables. Tables 5, 6, and 7 present the differences among the main variables according to socio-demographic variables using the Mann-Whitney test, t-test, and ANOVA tests.

**Table 5:** Difference in QNWL according to socio-demographic characteristics by Mann–Whitney U test (N = 193).

Variable	Category	N	Median	95% CI	U	P value
Gender	Male Female	N1 = 66 N2 = 127	156 159	146–159 157–165	3556	0.04∗
Highest education Degree in nursing	BSN degrees Master's degrees	N1 = 166 N2 = 27	159 156	154–162 151–168	2203	0.88
Dependent children	No Yes	N1 = 58 N2 = 134	160 157	153–168 153–161	3580	0.38
Dependent adult	No Yes	N1 = 95 N2 = 98	157 159	152–161 154–165	4299	0.35
Rotating shifts	No Yes	N1 = 30 N2 = 163	158 159	154–170 153–161	2255	0.49
Certified in speciality Area	No Yes	N1 = 164 N2 = 29	157 162	153–161 152–171	2209	0.54
Additional compensations	No Yes	N1 = 189 N2 = 4	159 157	154–162 142–170	353	0.82

**Table 6:** Difference in the main variables according to sociodemographic characteristics by ANOVA test (N = 193).

Variable	Category	N	QNWL						Job satisfaction						Intent to leave					
			Mean	SD	Std. error	95% CI	F	P value	Mean	SD	Std. error	95% CI	F	P value	Mean	SD	Std. error	95% CI	F	P value
**Age**	<30 30–40 >40	62 105 26	159.3 158.1 158.3	28.8 25.0 18.7	3.66 2.44 3.68	152–166 153–162 150–165	0.048	0.95	100 99.7 99.9	22.9 20.1 19.8	2.9 1.9 3.9	94–105 95–103 91–108	0.008	0.992	3.3 3.7 3.2	1.23 1.24 1.33	0.15 0.12 0.26	3.0–3.7 3.4–3.9 2.6–3.7	2.4	0.08
**Marital status**	Single Married Divorced& widowed	44 143 6	160.5 157.8 160.3	30.7 24 17	4.6 2.0 7.1	151–169 153–161 141–178	0.204	0.815	100.6 100.0 91.3	24 19 22	3.6 1.6 9.3	93–107 96–103 67–115	0.52	0.591	3.2 3.6 3	1.1 1.2 1.5	0.17 0.10 0.7	2.9–3.6 3.4–3.8 1.0–4.9	1.49	0.21
**Experience in nursing as RN**	<5 5 to 10 >10	45 80 68	159 156 68	31 22 24	4.7 2.4 3.0	149–168 151–161 154–166	0.456	0.634	102 97.8 100	24 18 21	3.6 2.0 2.5	95–110 93–101 95–105	0.88	0.417	3.4 3.5 3.6	1.3 1.2 1.2	0.19 0.13 0.15	3.0–3.8 3.2–3.7 3.3–40	0.68	0.50
**Hospital type**	Governmental Private University	64 64 65	147 166 162	25 27 19	3.1 3.4 2.3	140–153 159–173 157–166	11.1	.000∗	93 106 100	22 19 18	2.8 2.4 2.2	87–98 101–111 95–104	6.56	.002∗	3.61 3.22 3.83	1.29 1.30 1.12	0.16 0.16 0.14	3.2–3.9 2.8–3.5 4.1	4.0	0.02∗
**Unit type**	Emergency Medical Surgical Adult ICU & CCU PICU & NICU Paediatric Obstetrics & Gynae. Operation	32 26 34 35 14 11 9 32	160 163 153 155 152 164 172 158	17 23 27 30 23 16 31 18	4.8 4.5 4.6 5.1 6.3 4.8 10.4 3.2	150–169 153–172 144–163 144–165 139–166 153–175 148–196 152–165	0.997	0.435	104 96 104 93 88 104 106 101	18 21 16 26 19 12 21 20	3.2 4.1 2.8 4.5 5.1 3.9 7.2 3.5	98–111 87–104 98–110 84–102 77–100 96–113 89–123 94–108	1.94	0.064	3.3 3.8 3.4 3.4 3.4 4.0 4.2 3.3	1.4 1.0 1.2 1.2 1.3 0.94 1.0 1.3	0.24 0.20 0.21 0.21 0.37 0.28 0.36 0.23	2.8–3.8 3.4–4.3 3.0–3.9 3.0–3.9 2.6–4.2 3.4–4.7 3.3–5.0 2.8–3.8	1.21	0.29
**Salary**	Lower than average Within average Higher than average	48 98 47	155 160 157	27 27 16	3.9 2.8 2.4	147–163 154–165 152–162	0.516	0.598	99 101 97	20 22 17	2.9 2.2 2.6	93–105 96–105 91–102	0.70	0.496	3.1 3.7 3.6	1.2 1.2 1.2	0.18 0.12 0.17	2.7–3.5 3.4–3.9 3.2–4.0	3.49	0.03∗

∗ Significant difference.

**Table 7:** Difference in job satisfaction and intent to leave according to sociodemographic characteristics by t test (N = 193).

Variable	Category	N	Job satisfaction						Intent to leave					
			Mean	SD	Std. error	95% CI	t	P value	Mean	SD	Std. error	95% CI	t	P value
Gender	Male Female	66 127	98 100	23 19	2.9 1.7	-8.9–3.5	-.84	0.19	3.2 3.6	1.3 1.1	0.1 0.1	-0.7_-0.3	-2.13	0.01∗
Highest education Degree in nursing	BSN degrees Master's degrees	166 27	100 93	21 16	1.6 3.2	-1.5–15	1.60	0.05∗	3.6 3.1	1.2 1.0	0.9 0.2	0.47–0.98	-1.98	0.02∗
Dependent children	No Yes	58 134	101 99	21 20	2.8 1.7	-4.3–8.5	0.65	0.25	3.3 3.6	1.2 1.2	0.1 0.1	-0.6–0.09	-1.5	0.07
Dependent adults	No Yes	95 98	100 99	20 21	2.1 2.1	-4.9–6.9	0.33	0.36	3.5 3.5	1.2 1.2	1.2 1.2	-0.3–0.3	-0.19	0.4
Rotating shifts	No Yes	30 163	101 99	17 21	3.1 1.6	-6.0–10.3	0.59	0.30	3.5 3.5	1.3 1.2	0.2 0.0	-6.0–0.4	-0.25	0.4
Certified in speciality area	No Yes	164 29	99.9 99.2	21 20	1.6 3.8	-7.5–9.0	0.17	0.43	3.5 3.4	1.2 1.2	0.0 0.2	-0.3_-0.6	0.49	0.3
Additional compensations	No Yes	189 4	99.9 96.0	20 37	1.5 18.6	-55.2–63.1	0.21	0.42	3.5 3.0	1.2 0.8	0.0 0.4	-0.6–1.8	0.8	0.18

∗ Significant difference.

QNWL, Job satisfaction, and Intent to leave.

### 3.7 The correlations between the main variables and socio-demographic and work-related variables

Table 4 presents the correlations between the main variables and sociodemographic and work-related variables, where the correlation was weak and significantly positive between the QNWL and both participants' gender and hospital type. There was a weak and significantly positive correlation between job satisfaction and both participants' education and hospital type. Weak and significantly negative correlations were found between intent to leave and gender, hospital type, and salary. However, there was a weak and significantly positive correlation with the participants’ education.

## 4 Discussion

In the current study, the QNWL level was moderate, which is similar to previous studies in India, Saudi Arabia, and Turkey [2, 32, 33]

RNs showed a moderate level of job satisfaction similar to [33, 38, 39].

The QNWL was significantly correlated with job satisfaction, which is congruent with previous studies [33, 35]. This is not surprising as the quality of work life is an indicator of how satisfied employees are with their jobs [40].

QNWL was correlated negatively and significantly with the intent to leave, probably, because

more than half of the RNs in this study did not intend to leave their jobs and had a moderate level of quality of work life; however, if there was a better chance at another organisation, they would leave their job. The same finding was reported in a study by Faraji et al. [41].

There was also a significant negative correlation between job satisfaction and the intent to leave.

This result is congruent with other studies [18, 39, 42, 45, 46], as low job satisfaction is a contributing factor to nurses leaving their present jobs and professions [18, 47].

A previous study reported contradictory results regarding QNWL scores in relation to sex [37]. In the current study, females reported better QNWL scores than males, which may be explained by the fact that female nurses outnumber male nurses in hospitals, which is consistent with other studies [27, 34, 36].

Hospital conditions may refer to the hospital's size and policies, the type and number of patients, nurses' salaries and the physical environment, which have been shown to affect QNWL.

In a recent study, RNs were more satisfied working in private and university affiliated hospitals than in governmental hospitals, probably because nurses in non-governmental hospitals perform only the duties assigned in their job description.

Consistent with the study by Faizin et al. [36], a significant negative correlation was observed in this study between job satisfaction and the highest degree held in nursing, perhaps because of the absence of greater benefits that accompany higher degrees.

Furthermore, intent to leave was correlated significantly with gender, salary, type of hospital and the highest degree held in nursing. With respect to gender, men expressed a greater intention to leave their jobs, which was also reported in a previous Jordanian study conducted by Al Momani [42], possibly because men are the main breadwinners in their families; therefore, they may choose to leave for better offers.

Undoubtedly, salary is one of the main factors behind nurses' intention to leave Jordan, especially amidst the financial crises that have led to a drop in the Jordanian currency's purchasing power, which in turn has forced many nurses to search for better employment opportunities.

Hence, it was not surprising to find that the greatest intent to leave was among RNs making lower than the average salary [42]. Interestingly, another meta-analysis based on 106 primary studies conducted by Nei et al. [43] reported that there was no correlation between intent to leave and salary.

Additionally, RNs in the government had a higher intention to leave, followed by those in universities and private hospitals. Indeed, many factors can impact nurses' intent to leave an organisation, as reported by AlZamel in his integrative review [44], such as low salaries, lack of justice among nursing staff, bosses’ bullying of subordinates, work pressure, burnout, lack of career advancement opportunities, and lack of rewards and recognition [45]. It is worth mentioning that all of the previous factors apply more to governmental hospitals than to other types in Jordan.

Finally, the negative correlation between intent to leave and the highest degree of nursing in this study is congruent with a previous study by Kagwe [45], where it was reported that low job satisfaction and intent to leave seem to be mainly driven by the absence of extra rewards for nurses who earn higher degrees and certifications.

### 4.1 Study limitations

This study utilized a fairly long questionnaire composed of three parts, which may take a long time for the participants to complete. This may also involve reporting bias and providing socially desirable responses, which in turn may affect the interpretation of the results. Since convenience sampling, rather than random sampling, was employed, this might have created a certain degree of sampling error and thus affected the generalizability of the results. Moreover, the present study used a cross-sectional design instead of a longitudinal one, which hindered the ability to establish cause and effect relationships between the study variables [48]. Furthermore, use of only one method for variable assessment limits an in-depth understanding of the phenomena. Hence, applying qualitative research to address QNWL, job satisfaction, and the intent to leave the participants’ perceptions is strongly recommended.

## 5 Conclusion

This study revealed numerous aspects that contribute to reduced QNWL and job satisfaction for RNs and concluded that RNs in targeted hospitals had moderate levels on the QNWL scale and its dimensions. Therefore, we recommend that health service facilities consider the QNWL scale to help identify their nurses’ level of satisfaction and predict their intent to leave, and that nurse managers consider and manage these aspects.

Moreover, the present findings may enhance researchers’ awareness and prompt them to conduct further studies to fill the gap in research related to this topic.

## 6 Recommendations

Key suggestions are proposed to enhance QNWL among RNs based on the results of the current study:

1 Nursing managers should consider the family aspect of their nurses in terms of providing adequate working hours and sufficient vacations to achieve work-life balance.
2 Nursing managers and leaders must enable RNs to continue their education and develop their knowledge and skills in the nursing field by establishing partnerships with relevant educational organizations to provide distance learning and part-time opportunities.
3 Equitable distribution for the nursing workforce should be conducted to minimize workload and ensure high quality nursing care.
4 Communicating with the relevant authorities in order to appoint RNs to face the shortage.
5 Nurses' salaries should be increased in proportion to the tasks they perform.
6 Nurses should be provided with a private break area to keep their belongings in a safe place and take some rest.
7 Show the vital role of nurses in community care as well as in providing healthcare services and promoting population health.
8 It is the responsibility of hospital administrators and policymakers to provide a systematic career ladder to encourage nurses' career growth.
9 Nursing managers and leaders should provide support and opportunities to staff to enhance their independence in making decisions related to patient care.

### 6.1 Implications for nursing management

The findings of this study add to the existing body of knowledge and provide results that will benefit patients, nurses, and the healthcare organizations. Additionally, nurse managers will become knowledgeable about the factors that influence QNWL. Furthermore, they will be aware of current nurses' intention to leave their organizations. In turn, this will help nurse managers, healthcare leaders, and policymakers to plan and implement proper strategies to improve work environments for nurses to retain them and attract more qualified nurses. Such developments are expected to increase job satisfaction, enhance individual and organizational performance, improve home and job environments, increase nurses’ commitment, and attract and retain RNs.

## Declarations

### Author contribution statement

Muna Salahat & Zaid Al-Hamdan: Conceived and designed the experiments; Performed the experiments; Analyzed and interpreted the data; Contributed reagents, materials, analysis tools or data; Wrote the paper.

### Funding statement

This research did not receive any specific grant from funding agencies in the public, commercial, or not-for-profit sectors.

### Data availability statement

Data included in article/supp. material/referenced in article.

### Declaration of interests statement

The authors declare no conflict of interest.

### Additional information

No additional information is available for this paper.
